# TGF-β induces EMT in thyroid cancer cells by regulating transcription factors

**DOI:** 10.1186/s13044-025-00243-w

**Published:** 2025-06-10

**Authors:** Jianjian Xiang, Nannan Lv, Shanyu Yin, Tong Zhao, Fei Liu, Lan Cheng, Feng Liu, Jinsong Kuang

**Affiliations:** 1 Department of Ultrasound Medicine, First Affiliated Hospital, Zhejiang University School of Medicine, Hangzhou, 310000 China; 2 Department of Endocrinology, The Fourth People’s Hospital of Shenyang, China Medical University, 20 Huanghe South Street, Huanggu District, Shenyang, 10032 China; 3 Department of Endocrinology, First Affiliated Hospital, Zhejiang University School of Medicine, Hangzhou, 310000 China

**Keywords:** Thyroid carcinoma, Epithelial–mesenchymal transition, Transforming growth factor beta, Thyroid stimulating hormone, Transcription factor

## Abstract

**Background:**

Transforming growth factor-β (TGF-β) plays well-established roles in cancer cell invasion and epithelial–mesenchymal transition (EMT); however, its role in thyroid carcinoma (TC) remains unclear. This study aimed to evaluate the effects of TGF-β on EMT in TC and determine its underlying mechanisms.

**Methods:**

Treatment of TC cell lines with TGF-β the morphology of thyroid cancer cells changed, Immunofluorescence staining revealed that the localization of E-cadherin shifted from the cell membrane to the cytoplasm, and the fluorescence intensity decreases. Wound-healing assay in BCPAP and TPC-1 revealed that migration ability was significantly higher in the TGF-β (5 ng/mL) treatment group than in the control group (*P* < 0.01).

**Results:**

Transwell assays showed that the invasive abilities of TGF-β-treated BCPAP, TPC-1, and K1 cells were 7-, 10-, and 6-fold higher than those of the control group, respectively (*P* < 0.05). After TGF-β treatment, mRNA levels of SNAI1 significantly increased in TPC-1 and BCPAP cell lines. Treatment of TC cell lines with TGF-β downregulated the epithelial marker E-cadherin and upregulated the mesenchymal markers N-cadherin and vimentin, at the mRNA level. Western blotting indicated similar results at the protein level, TSH could enhance this process.

**Conclusions:**

TGF-β promotes EMT-like phenotypic changes in thyroid cancer cells, accompanied by upregulation of SNAI1 and EMT-related markers, which is enhanced by TSH. Overall, this study provides a basis for the development of therapeutic strategies for TC targeting the EMT.

## Background

In 2018, approximately 567,000 new cases of thyroid cancer (TC) were reported worldwide, making it the ninth most common form of cancer. The incidence rate of TC in women is 3 times that of men, and TC accounts for 5.1% of the total cancer burden in women [1]. In China, TC ranks fourth in incidence among malignancies in women, and the population aged 30 years and below accounts for the highest number of new cases [2]. Other epidemiological data have also demonstrated that TC is characterized by a female predominance, increasingly younger age of onset, and continuous rise in mortality rate with age [3–5]. Papillary thyroid carcinoma (PTC) is the most common histological type of TC, accounting for approximately 85% of all TC cases. In the United States, the overall mortality rates for TC and advanced TC have increased significantly from 1.1%/year in 1994 to 2.9%/year in 2013, with approximately one-third of the observed increase in mortality explained by advanced PTC [6].

The invasive and metastatic behaviors of cancer are related not only to the genetic characteristics of tumor cells but also to their environment, a concept known as the tumor microenvironment (TME). Cytokines, the extracellular matrix (ECM), hormones, and viruses are key components of the TME [7]. Tumor cells and their associated leukocytes and/or platelets can produce inflammatory mediators, which promote invasion and metastasis [8, 9]. Transforming growth factors (TGFs) are key cytokines involved in various cellular activities, such as cell growth, differentiation, motility, invasion, and apoptosis. Although TGFs are dynamically regulated and participate in the maintenance of tissue homeostasis, they often exhibit prolonged overexpression in disease states (including cancer, fibrosis, and inflammation) and affect disease progression through the regulation of cell growth and migration. Therefore, TGFs have become popular targets in drug development [10–12].

The epithelial–mesenchymal transition (EMT) is a complex process in which epithelial cells lose their epithelial characteristics and acquire a mesenchymal phenotype. It is characterized by the loss of expression of the cell-cell adhesion molecule E-cadherin and overexpression of the mesenchymal cell component N-cadherin, which confers migratory ability to cells. The EMT is involved in the invasion and metastasis of many types of tumors. Mutations and dedifferentiation of epithelial cells during tumor invasion and progression are now widely recognized as hallmarks of tumor progression [13, 14]. Furthermore, chronic stimulation with a low dose of TGF-β can induce the occurrence of EMT in various tumor cell lines in vitro, thus enhancing their invasive and metastatic capabilities [15–18].

In view of the continuous increase in the incidence of TC and the adverse consequences of its recurrence, the in-depth exploration of molecular mechanisms underlying the occurrence and development of autoimmune thyroid diseases and TC invasion and metastasis and the establishment of effective diagnostic and treatment methods are of immense practical significance.

As the role of TGF-β in TC is not clearly established, the present study aimed to confirm the critical role of TGF-β in the invasion, metastasis, and EMT of TC and to identify the potential molecular mechanisms involved. We utilized TGF-β to induce the EMT in human TC cell lines and observed the expression of SNAI1, SNAI2, ZEB1, TWIST during this process. Previous studies have reported controversial results over the potential increase in thyroid cancer risk due to TSH [19–21] and iodine intake [22, 23]. Therefore, we also observed the combined effects of TGF-β and TSH or iodine in thyroid cancer cells. The findings of this study can serve as a reference for further research aimed at monitoring TC invasion and metastasis and for the development of therapeutic targets and methods.

## Methods

### Cell lines

This study was conducted in accordance to already-published protocols [24]. Specifically, human thyroid carcinoma cell lines TPC-1 (RET/PTC1 rearrangement), BCPAP (BRAF V600E mutation), and K1 (RAS mutation) were used. The different cell lines were acquired as follows: TPC-1 was provided by Dr. Haugen (Division of Endocrinology, Diabetes, and Metabolism, University of Colorado Denver); BCPAP was purchased from the Leibniz Institute DSMZ-German Collection of Microorganisms and Cell Cultures (Braunschweig, Germany); and K1 was obtained from the Health Protection Agency Culture Collections (Salisbury, UK). All the cell lines were tested for mycoplasma contamination and authenticated using STR profiling prior to use. TPC-1 cells were cultured in high-glucose DMEM, whereas BCPAP and K1 cells were maintained in RPMI-1640 medium containing 2 mmol/L L-glutamine (Gibco). All media were supplemented with 10% fetal bovine serum (FBS, Gibco). The cells were cultured at 37 °C in a humidified atmosphere containing 5% CO_2_.

### Immunofluorescence

Cover slips were placed in the wells of a six-well plate containing cells and treated with TGF-β (10 ng/mL) for 36 h. When the cells reached 95–100% confluence, they were removed from the incubator, and the culture medium was discarded. The cells were fixed on ice using 4% paraformaldehyde for 20 min, then treated with 0.1% Triton X-100 at room temperature for 5 min, followed by blocking with 1% BSA for 30 min. The samples were then incubated overnight at 4 °C with primary antibodies against E-cadherin (1:200, SC-8426, Santa Cruz), N-cadherin (1:200, SC-8424,Santa Cruz), and vimentin (1:50, SC-66002, Santa Cruz). After incubation, the samples were treated with Alexa secondary antibodies (1:400; Invitrogen) at room temperature for 1 h in the dark. Following washes, the cells were finally stained with DAPI (Invitrogen) and stored in the dark at 4 °C for subsequent microscopic examination.

### Wound healing assay

After treating the cells with TGF-β (Invitrogen, 5 ng/mL)for 36 h, the cells were digested and subsequently seeded into a 12-well plate, ensuring that each well was adequately covered with cells. A 10-µL pipette tip was used to create a consistent scratch in the center of each well. The wells were then washed three times with PBS to remove cell debris generated from the scratching. The cells were cultured in serum-free medium and incubated, with images captured every 4–6 h for documentation. The experimental outcomes were then extracted from the captured images and analyzed.

### Cell migration and invasion assays

The surface of the upper chamber of Costar Transwell chambers (8 μm pore size) was coated with 50 mg/L Matrigel (1:7 dilution) and allowed to dry at 4 °C. After removing the residual liquid, the coated surface was hydrated with 50 µL of serum-free medium containing 10 g/L BSA at 37 °C for 30 min. Following treatment with TGF-β (5 ng/mL) and various pathway inhibitors (Sigma) for 36 h, a cell suspension was subsequently prepared. After digestion, the cells were resuspended in serum-free medium containing BSA, with the cell density adjusted to 5 × 10^5^ cells/mL. Approximately 200 µL of the cell suspension was then added to the upper chamber, whereas 500 µL of normal culture medium was added to the lower chamber. After 36 h of culture, Matrigel and any cells in the chamber were gently wiped away with a damp cotton swab, and the upper chamber was carefully removed and identified. The cells were then fixed with 4% formaldehyde and stained with hematoxylin and eosin. Six randomly selected fields were observed for cells attached to the underside of the membrane using a high-power microscope (×400). This experimental procedure was repeated thrice.

### RNA extraction and qRT-PCR

Total RNA was extracted using TRIzol reagent (Invitrogen, Life Technologies, Grand Island, NY, USA) and reverse transcribed using the PrimeScript™ RT reagent Kit (TaKaRa). The obtained cDNA was analyzed in triplicate using SYBR^®^ Premix Ex Taq™ (TaKaRa). The relative mRNA concentration was calculated using the formula 2−(Ct–Cc), where Ct and Cc denote the average threshold cycle differences normalized to GAPDH values.

RT-PCR primer sequences for monitoring mRNA expression were: E-cadherin (CDH1) forward: 5′-TGCCCAGAAAATGAAAAAGG-3′, E-cadherin (CDH1) reverse: 5′-GTGTATGTGGCAA TGCGTTC-3′; N-cadherin (CDH2) forward: 5′-ACAGTGGCCACCTACAAA GG-3′, N-cadherin (CDH2) reverse: 5′-CCGAGATGGGGTTGATAATG-3′; Vimentin forward: 5′-GAGAACTTTGC CGTTGAAGC-3′, Vimentin reverse: 5′-GCTTCCTGTAG GTGGCAATC-3′; SNAI1 (SNAIL) forward: 5′-CCTCCCTGTCAGATGAGGA C-3′, SNAI1 (SNAIL) reverse: 5′-CCAGGCTGAGGTAT TCCTTG-3′; SNAI2 (SLUG) forward: 5′-TTCGGACCCACACATT ACCT-3′, SNAI2 (SLUG) reverse: 5′-GCAGTGAGGGCAAGAAAAA G-3′; TWIST1 forward: 5′-GGAGTC CGCAGTCTTA CGAG-3′, TWIST1 reverse: 5′-TCTGGAGGACCTG GTAGAGG-3′; ZEB1 forward: 5′-GATGAT GAATGCGAGTCAGATGC-3′, ZEB1 reverse: 5′-ACAGCAGTGTCT TGTTGTTGTAG-3′; and GAPDH forward: 5′-ACCCAGAAGACTG TGGATGG-3′, GAPDH reverse: 5′-TCTAGACGGCA GGTCAGGTC-3′.

### Western blot assay

After washing the cells twice with ice-cold PBS, the cells were lysed on ice using RIPA buffer (Sigma-Aldrich, St. Louis, MO), followed by protein quantification using the QuantiPro BCA Protein Assay Kit (Sigma-Aldrich). The proteins were denatured with sample buffer at 100 °C for 5 min. Equal amounts of protein (50 µg) were separated according to molecular weight through 10–12% SDS-PAGE gel electrophoresis. The separated proteins were then transferred to PVDF membranes (Bio-Rad) and blocked with blocking buffer (5% non-fat milk in TBS containing 0.1% Tween 20) for 2 h. The membranes were then incubated overnight at 4 °C with primary antibodies. Vimentin (SC-66002, Santa Cruz), E-cadherin(SC-8426, Santa Cruz), N-cadherin (SC-8424, Santa Cruz), and β-actin primary(SC-17582, Santa Cruz) antibodies were purchased from Santa Cruz Biotechnology, all at a 1:1000 dilution. After washing, the membranes were incubated at room temperature for 1.5 h with horseradish peroxidase-conjugated secondary antibodies (Santa Cruz). The blots were developed using the ECL Western Blotting Detection System (Pierce) on the FluorChem^®^ FC2 (Alpha Innotech, CA, USA), and quantified using ImageJ^®^ software.

### Statistical analysis

All statistical analyses were performed using SPSS 20 software. Count data were expressed as the mean ± standard error of the mean (SEM). Differences between two groups were compared using the T-test, while differences among three groups were assessed using one-way ANOVA. For the comparison of differences between the treatment group and the control group, Dunnett’s analysis was employed. A p-value of less than 0.05 was considered statistically significant.

## RESULTS

### Morphological and the localization of EMT markers changes in PTC cell lines after TGF-β treatment

**Fig. 1:**
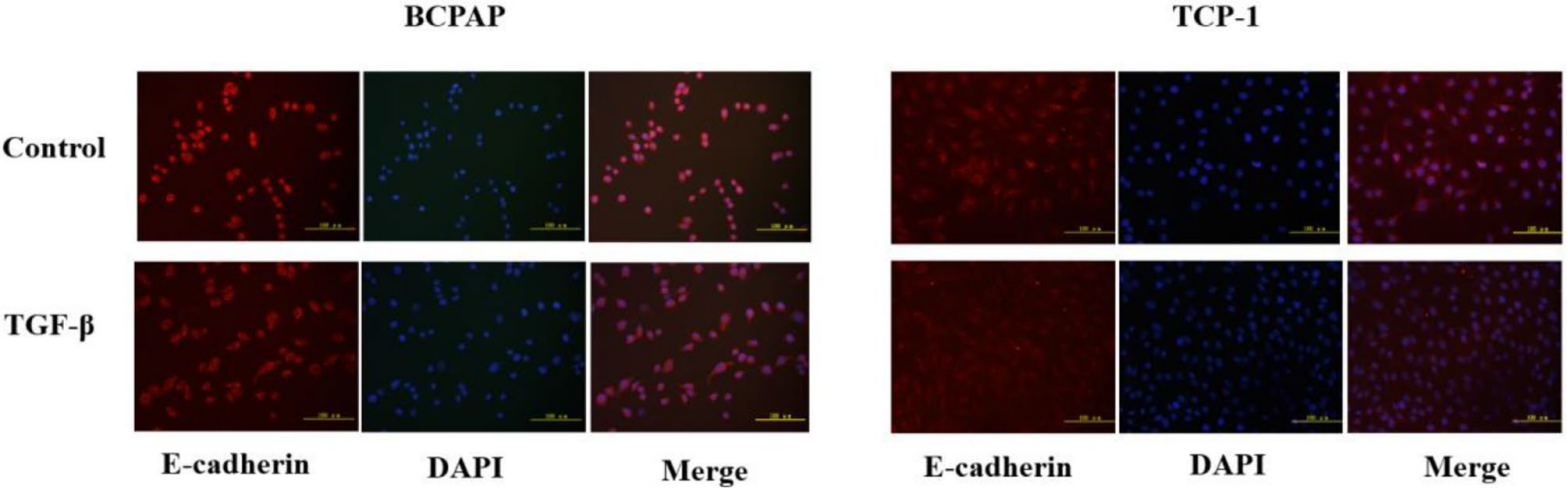
Effects of TGF-β on the localization of the EMT marker protein E-cadherin in the BCPAP and TPC-1 cell lines. Cells were treated with TGF-β (10 ng/mL) for 36 h. Expression of E-cadherin (red) was analyzed by immunofluorescence staining. Nuclei were visualized with DAPI staining (blue). Magnification: × 200; scale bars: 100 μm

TGF-β treatment for 36 h led to a gradual change in TPC-1 morphology from a plump, round shape to a spindle shape, which allowed the cells to move more easily. Similar morphological changes were observed in BCPAP, suggesting that the cells were undergoing EMT. Immuno- fluorescence staining of cells revealed that E-cadherin in the three TC cell lines was primarily localized to the cell membrane before treatment. Following treatment with 10 ng/mL TGF-β for 36 h, E-cadherin in all cell lines became primarily localized to the cytoplasm, along with a decrease in expression (Figs. 1).

### Changes in the migratory ability of TC cells after TGF-β treatment

**Fig. 2:**
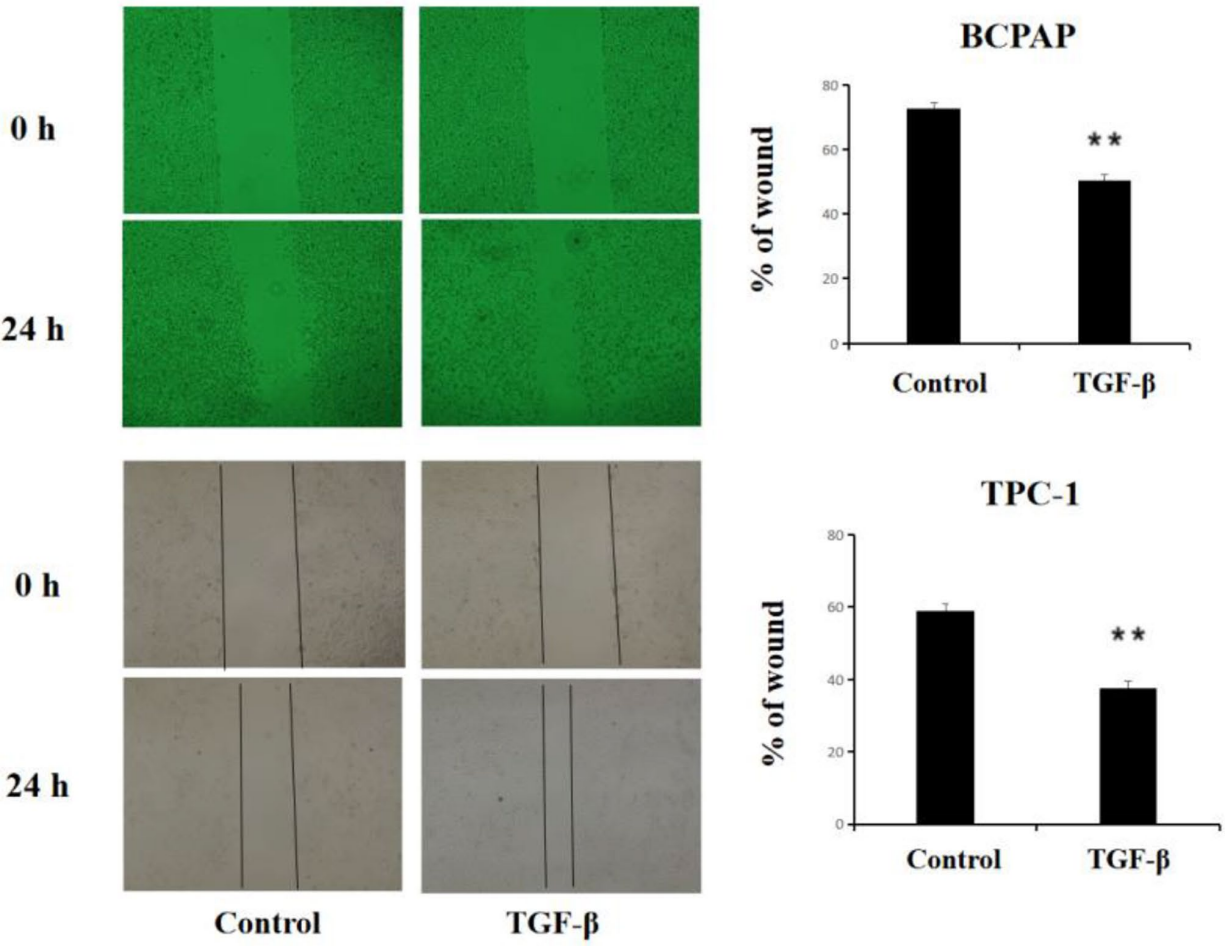
Effects of TGF-β on the migratory ability of BCPAP and TPC-1 cells. cells migration ability were detected using wound healing assays after pretreatment with TGF-β (5 ng/mL) for 36 h. The results are representative of at least three independent experiments. Error bars represent SEM. **P < 0.01

A wound-healing assay was performed after treatment with TGF-β (5 ng/mL) for 36 h. After 24 h of migration, the BCPAP and TPC-1 cell lines in the treatment group exhibited higher rates of migration than those in the control group (*P* < 0.01), as shown in Figs. 2. Due to the classification of K1 cells (RAS-mutant) as a rare mutation subtype, the localization of EMT markers and migratory phenotypes were not analyzed.

### Changes in invasive and metastatic activities of TC cells after TGF-β treatment

**Fig. 3:**
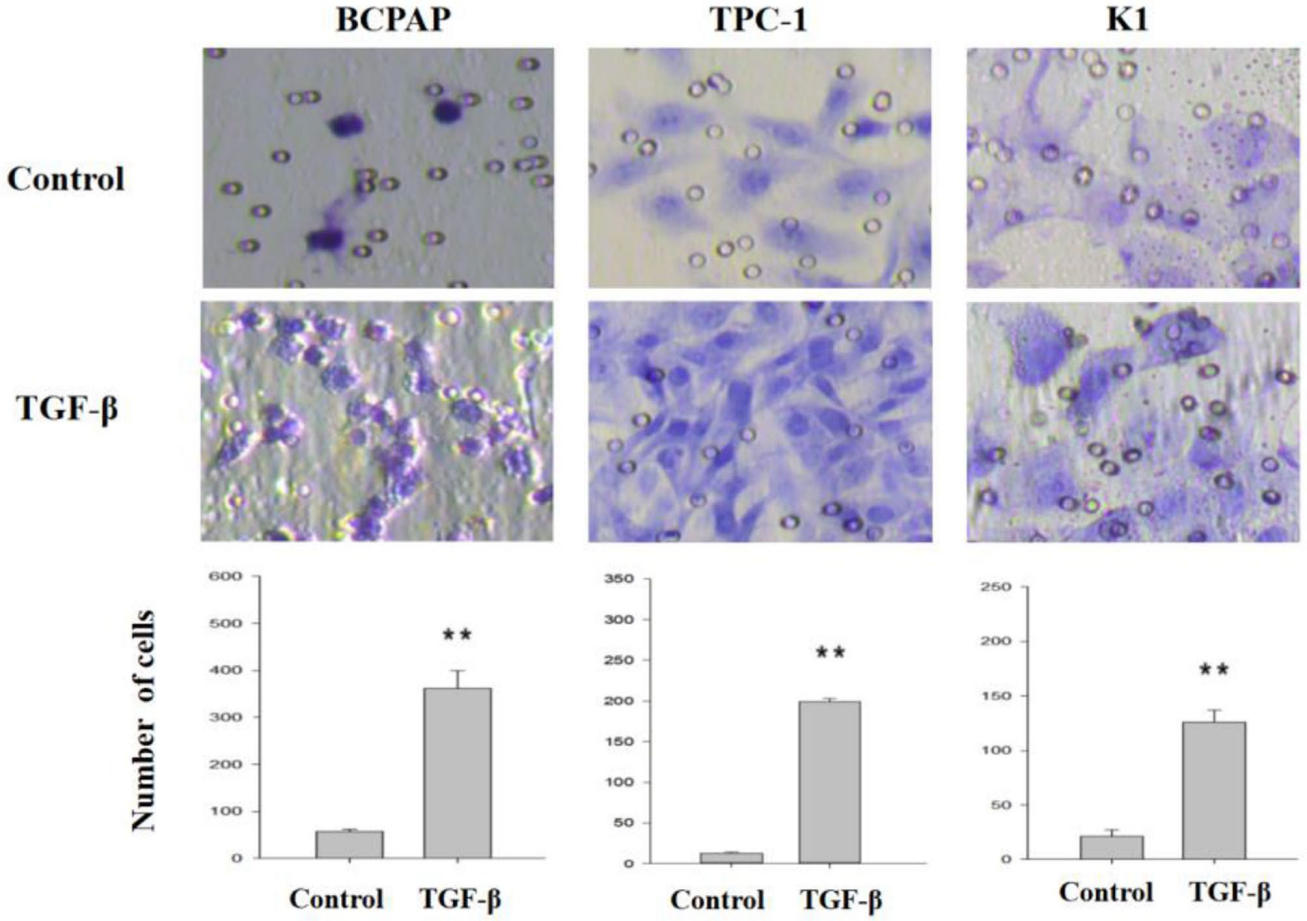
Effects of TGF-β on invasive and metastatic activities of cells. Invasion capacity of papillary thyroid cancer cell lines was detected using transwell assays after treatment with TGF-β (5 ng/mL) for 36 h. The results are representative of at least three independent experiments. Error bars represent SEM. **P < 0.01

After treatment with TGF-β for 36 h, cells were digested, suspended, transferred into a Transwell chamber, and cultured for 36 h. Cells that migrated through the Matrigel were counted (see the Experimental Methods section for the detailed experimental procedure). As shown in Fig. 3, the invasive and metastatic activities of BCPAP, TPC-1, and K1 cells treated with TGF-β were 7-, 10-, and 6-fold higher than those in the control group, and the differences were statistically significant.

### Increased expression of transcription factors during TGF-β-induced EMT

**Fig. 4:**
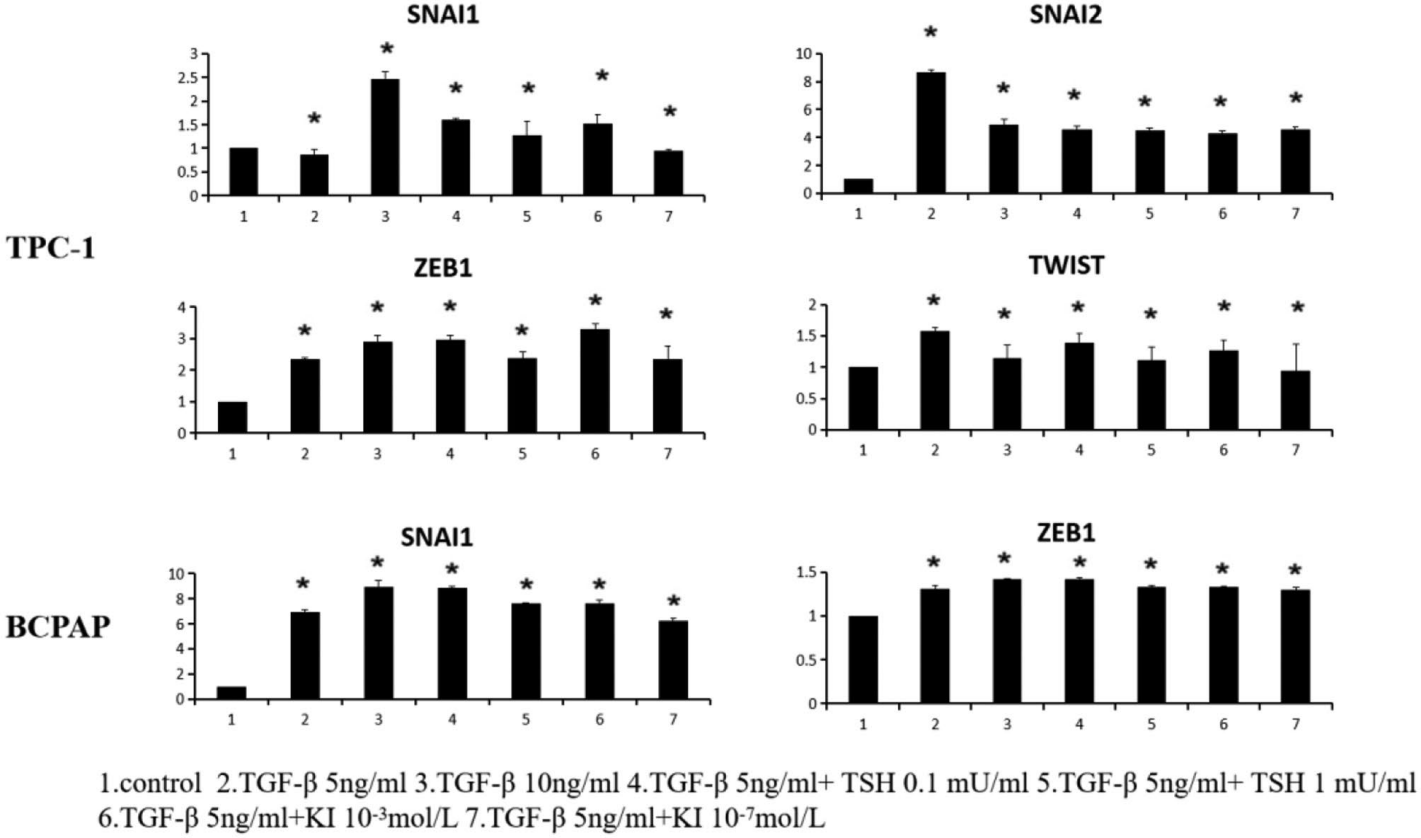
Influence of TGF-β treatment on transcription factor expression in TCP-1 cells. Cancer cells were serum-starved for 24 h before treatments, then treated with different concentrations of TGF-β, TGF-β + TSH, and TGF-β + KI for 12 h. Total RNA was isolated and subjected to qRT-PCR and values were normalized with GAPDH used as an internal control. Results are reported as mean of three independent experiments. Columns, mean (*n* = 3); bars, SEM. *P < 0.05

The results described above clearly demonstrated that TGF-β can induce the EMT in TC cell lines. Our previous research has suggested that the NF-κB signaling pathway has a crucial role in TNF-α-induced EMT, invasion, and metastasis in TC via the regulation of TWIST1 expression [25]. To determine whether transcription factors play a critical role in the EMT of TC, we measured gene and protein expression levels of transcription factors in TC cells after treatment with TGF-β. We utilized three representative TC cell lines, namely TPC-1 (reported to harbor RET/PTC1 rearrangement), BCPA (reported to harbor BRAF V600E mutations), to evaluate the effect of different mutations on transcription factor expression. The results are summarized in Fig. 4. As determined by using polymerase chain reaction (PCR), after 12 h of TGF-β treatment, the mRNA level of *SNAI1* was higher than that in the control for all three cell lines. The highest increases were approximately 2.5-fold in TPC-1 (Fig. 4), 9.0-fold in BCPAP, and these differences were statistically significant.

**Fig. 5:**
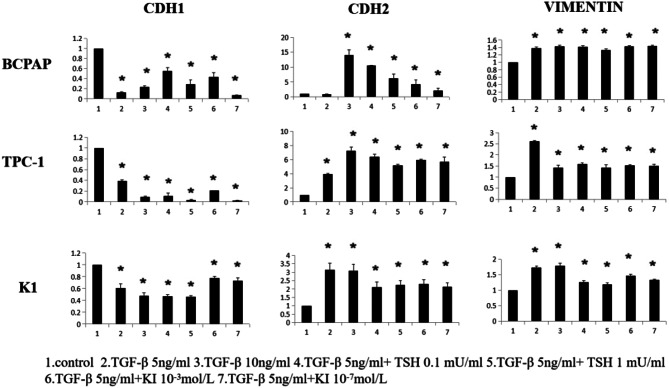
Influence of TGF-β treatment on mRNA expression levels of EMT markers in TC cell lines. Cancer cells were serum-starved for 24 h before treatments, then treated with different concentrations of TGF-β, TGF-β + TSH, and TGF-β + KI for 12 h. Total RNA was isolated and subjected to qRT-PCR, values were normalized with GAPDH used as an internal control. Results are reported as mean ± SEM of three independent experiments; Columns, mean (*n* = 3); bars, SEM. *P < 0.05

As shown in Fig. 5, following stimulation with TGF-β, levels of *SNAI2*,* ZEB1*, and *TWIST1* were also elevated in TPC-1.In the BCPAP cell line, there was an increase in the expression level of *SNAI1* after TGF-β stimulation. *ZEB1* expression also showed an increase; however, the difference was not statistically significant. These results suggest that the mechanisms underlying TGF-β-induced EMT may differ across TC cell lines with different mutations. However, further research is required to confirm this conjecture.

### Influence of TGF-β treatment on mRNA levels of EMT markers in TC cell lines

The TC cell lines BCPAP, TPC-1, and K1 were stimulated with different concentrations of TGF-β (0, 5, and 10 ng/mL combined with thyroid-stimulating hormone (TSH) (0.1 and 1 mU/mL) and potassium iodide (KI) (10^− 3^ and 10^− 7^ mol/L). After 12 h of treatment, cell precipitates were collected for RNA extraction and analyses of mRNA levels of the canonical EMT markers E-cadherin, N-cadherin, and vimentin.

As shown in Fig. 5, treatment with TGF-β alone and in combination with TSH and KI decreased the mRNA level of the epithelial marker E-cadherin and increased the mRNA levels of the mesenchymal markers N-cadherin and vimentin. In BCPAP, the mRNA level of E-cadherin was decreased by approximately 60–90% (*P* < 0.05). The mRNA level of N-cadherin increased by up to 14-fold in the treatment groups after 12 h (*P* < 0.05). However, the increase in vimentin mRNA levels was less substantial (i.e., 1.3- to 1.4-fold; *P* < 0.05).

In TPC-1 cells, E-cadherin levels were approximately 70–90% lower in the treatment groups than in the control group (*P* = 0.05). The mRNA level of N-cadherin was 3- to 6-fold higher in the treatment groups than in the control group (*P* < 0.05). Moreover, the addition of TSH enhanced these effects. Vimentin mRNA levels also increase, to a smaller extent, with the treatment groups showing increments of 1.4- to 2.6-fold (*P* < 0.05).

In the K1 cell line, the mRNA level of E-cadherin was approximately 30–60% lower in the treatment groups than in the control group (*P* < 0.05). The mRNA level of N-cadherin was 2- to 3-fold higher in the treatment groups (*P* < 0.05). The increase in vimentin mRNA levels was smaller with the various treatment groups exhibiting increases of 1.2- to 1.7-fold (*P* < 0.05).

### Influence of TGF-β treatment on protein levels of EMT markers in TC cell lines

The TC cell lines BCPAP and TPC-1 were treated with different concentrations of TGF-β (0, 5, and 10 ng/mL) combined with tumor necrosis factor-alpha (TNF-α) (10 ng/mL), TSH (0.1 and 1 mU/mL), and KI (10^− 3^ and 10^− 7^ mol/L). After 36 h of treatment, the cell precipitates were collected for total protein extraction to measure the protein levels of canonical EMT markers.

**Fig. 6:**
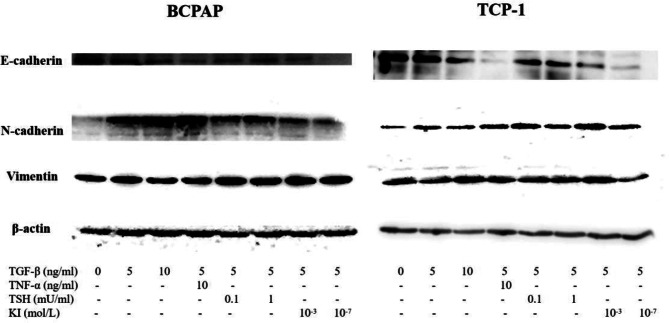
Influence of TGF-β treatment on protein levels of EMT markers in TC cell lines. Human thyroid carcinoma cell lines BCPAP and TPC-1 were used. Cancer cells were serum-starved for 24 h before treatments. EMT markers E-cadherin, N-cadherin and vimentin were detected. Total protein was isolated and subjected to Western blot, expression of β-action served as a loading control. Results are reported as mean of three independent experiments

As shown in Fig. 6, western blotting revealed that 36 h of treatment with different TGF-β concentrations led to the downregulation of E-cadherin and upregulation of N-cadherin and vimentin in the BCPAP cell line. E-cadherin protein levels were approximately 57–76% lower in the various TGF-β treatment groups than in the control group, while N-cadherin protein levels were 1.77- to 7.23-fold higher, with the greatest increase occurring in the TGF-β + TNF-α group. The protein levels of vimentin were 1.06- to 1.22-fold higher in the TGF-β treatment groups than in the control group and also showed the greatest increase in the TGF-β + TNF-α group. The differences described above were statistically significant.

In the TPC-1 cell line, decreases in E-cadherin protein levels and increases in N-cadherin and vimentin protein levels were also observed after TGF-β treatment. E-cadherin protein level decreased by approximately 8–85% in the TGF-β treatment groups. N-cadherin protein levels increased by 1.95- to 14.77-fold. Addition of TSH also enhanced these effects, similar to that in gene expression. Moreover, vimentin protein levels increased by 1.16- to 2.28-fold. The differences described above were statistically significant.

## Discussion

This study established the role of TGF-β in the epithelial–mesenchymal transition (EMT) and cell migration and invasion in PTC. Furthermore, we observed differences in the effects of TGF-β among three PTC cell lines, indicating the importance of genotype/mutation status. It was also found that transcription factor responses differed in cell lines with different mutations, and for the first time, it was discovered that TSH and TGF had a synergistic effect when inducing EMT in some cell lines.

The process by which normal cells are converted to cancer cells is known as carcinogenesis. Only a small proportion of cancers are caused by germline mutations, whereas the majority (90%) are caused by somatic mutations and environmental factors. Many environmental carcinogenic factors and risk factors are associated with the development of chronic inflammation [26]. Tumor metastasis refers to the process by which malignant tumor cells spread from the primary site to other sites via lymphatic vessels, blood vessels, or body cavities and continue to grow at the secondary sites. The metastatic process can be roughly divided into four steps. In the first step, represented by the EMT, cancer cells acquire fibrous characteristics that increase their motility and enable them to reach blood vessels or lymphatic vessels by crossing the basement membrane [13]. During the second step, cancer cells enter and survive in blood vessels and lymphatic vessels. The third step involves the escape of cancer cells from blood vessels and their colonization of distant sites. Inflammation promotes the aforementioned steps through the production of substances that increase vascular permeability. From a clinical perspective, more than 90% of patients with cancer die from metastases. Although the majority of mechanisms contributing to the metastatic process remain unclear, the joint action of cancer cells, immune cells, inflammatory cells, and stromal components is paramount [27].

Research on the biological functions of TGF-β was initially focused mainly on inflammation, tissue repair, and embryonic development. In recent years, researchers have found that TGF-β also exerts significant regulatory effects on the growth, differentiation, and immune functions of cells. TGF-β is one of the first EMT inducers discovered in cancer and serves as a key regulator of cancer cell progression and metastasis [28]. Studies have revealed that human thyroid tissue can express genes encoding inflammatory proteins, such as CXCR4, CD44, OPN, CXCL1, CXCL10, and SDF-1. Activated proto-oncogenes, such as *RET/PTC*, *RAS*, and *BRAF*, in thyroid tumors can trigger mitogen-activated protein kinase (MAPK) cascades, thereby promoting the spontaneous transformation of cells into a proinflammatory phenotype. This consequently leads to increased expression of certain cytokines, chemokines, and their receptors [29, 30]. A study by Nucera [31] suggested that *BRAF* V600E mutations and the TME in PTC are associated with the EMT process, which promotes tumor proliferation. In the present study, we investigated the influence of the proinflammatory cytokine TGF-β on the malignant progression of PTC cells in vitro. We found that TGF-β enhanced the migratory and invasive abilities of cells, accompanied by changes in indicators of the EMT, i.e., the downregulation of E-cadherin, upregulation of N-cadherin and vimentin, and changes in cell morphology. The three PTC cell lines TPC-1, BCPAP, and K1 exhibited similar responses to TGF-β. This suggests that proto-oncogene mutations do not affect the activity of TGF-β, at least in terms of equivalent changes at the level of E-cadherin expression. However, further research is required to determine whether such mutations exert influences on different signaling pathways and other factors that may induce EMT.

However, we found that TSH synergistically enhanced TGF-β-induced EMT in RET mutated thyroid cell lines (TPC-1) on both mRNA and protein levels, but only synergistically enhanced TGF-β-induced EMT in E-cadherin and N-cadherin, as shown in Figs. 6 and 7. These results differ from those reported by Orim F et al. [19], who suggested that thyroid cancer induced by BRAFV600E in mice was more invasive when stimulated by TSH.

Our results showed that E-cadherin is highly expressed in untreated PTC cells, in agreement with the results of Liu et al., indicating that E-cadherin is expressed in well-differentiated TCs (e.g., PTC and follicular thyroid carcinoma) but not in undifferentiated TCs [32]. It has also been found that E-cadherin expression is downregulated in the undifferentiated TC cell line WRO upon TNF-α stimulation and returns to normal after the removal of TNF-α [33]. Researchers have also reported that the downregulation of E-cadherin in well-differentiated TCs is associated with a poor prognosis [34].

Transcription factors are regarded as upstream regulatory factors of the EMT. Members of the Snail family of transcriptional repressors (e.g., SNAIL and SLUG) and other repressors of E-cadherin gene expression, such as ZEB1, ZEB2, and TWIST1, have been detected at EMT sites at the leading edge of metastatic tumors [35]. These transcription factors are closely associated with cancer invasion and the TNM stage [36–38]. To investigate the mechanisms underlying TGF-β-induced EMT in PTC, we evaluated the expression levels of these previously characterized transcription factors. We detected differences in the expression of transcription factors among cell lines, as shown in Figs. 5. In particular, the gene expression level of Snail increased in response to TGF-β treatment in all three cell lines. Baquero et al. have also reported that the overexpression of Snail can induce the EMT and promote invasiveness in TC cells harboring *BRAF* mutations [39, 40]. However, the expression levels of *SLUG*,* TWIST*, and *ZEB* differed among cell lines, and this variation has not been reported previously in PTC cell lines. This suggests that genetic mutations in PTC may influence specific pathways related to EMT induction; however, further research is warranted to confirm this idea.

When confronted with TGF-β and TNF-α [25], thyroid cancer cells respond differently. Specifically, under the stimulation of TNF-α, the expression levels of SNAI1 and TWIST1 in BCPAC cells significantly increased. In contrast, under the influence of TGF, BCPAP cells demonstrated an elevated expression of SNAI1 and ZEB1, while the changes in other related factors, e.g., SNAI2, were negligible. Overall, this suggests that TNF-α and TGF-β are driven by different underlying mechanisms.

## Conclusions

In conclusion, TGF-β promotes EMT-like phenotypic changes in thyroid cancer cells, accompanied by upregulation of SNAI1 and EMT-related markers, which is enhanced by TSH. The expression of Snail is necessary for the EMT and the acquisition of a mesenchymal phenotype in PTC. The elucidation of molecular mechanisms linking Snail expression and EMT in TC will thus aid in the discovery of novel treatment strategies. Therefore, future research efforts, particularly in vivo studies, should focus on the roles of other pathways (STAT3 and AP-1) and regulatory factors (e.g., SLUG), as well as the effectiveness of TSH in the EMT.

## Data Availability

No datasets were generated or analysed during the current study.

## Abbreviations

BRAF: B-type Rapidly Accelerated Fibrosarcoma kinase
EMT: Epithelial mesenchymal transition
TC: Thyroid cancer
PTC: Papillary thyroid cancer
FTC: Follicular thyroid carcinoma
ATC: Anaplastic thyroid carcinoma
mRNA: Messenger RNA
RT-PCR: Real Time- Polymerase Chain Reaction
TNF-α: Tumor Necrosis Factor-α
IGF: Insulin-like Growth Factors
TGF-β: Transforming Growth Factor-β
MAPK: Mitogen-activated protein kinases
IFN: Interferon
TME: The tumor microenvironment
ECM: Extracellular matrix
TGFs: Transforming growth factors
